# Data-driven model discovery of ideal four-wave mixing in nonlinear fibre optics

**DOI:** 10.1038/s41598-022-16586-5

**Published:** 2022-07-26

**Authors:** Andrei V. Ermolaev, Anastasiia Sheveleva, Goëry Genty, Christophe Finot, John M. Dudley

**Affiliations:** 1 Institut FEMTO-ST, Université Bourgogne Franche-Comté CNRS UMR 6174, 25000 Besançon, France; 2 Laboratoire Interdisciplinaire Carnot de Bourgogne, Université Bourgogne Franche-Comté CNRS UMR 6303, 21078 Dijon, France; 3 Photonics Laboratory, Tampere University, FI-33104 Tampere, Finland

**Keywords:** Fibre optics and optical communications, Nonlinear optics, Ultrafast photonics

## Abstract

We show using numerical simulations that data driven discovery using sparse regression can be used to extract the governing differential equation model of ideal four-wave mixing in a nonlinear Schrödinger equation optical fibre system. Specifically, we consider the evolution of a strong single frequency pump interacting with two frequency detuned sidebands where the dynamics are governed by a reduced Hamiltonian system describing pump-sideband coupling. Based only on generated dynamical data from this system, sparse regression successfully recovers the underlying physical model, fully capturing the dynamical landscape on both sides of the system separatrix. We also discuss how analysing an ensemble over different initial conditions allows us to reliably identify the governing model in the presence of noise. These results extend the use of data driven discovery to ideal four-wave mixing in nonlinear Schrödinger equation systems.

## Introduction

The tools and methods of machine learning (ML) are driving a revolution in the understanding of complex dynamics^1–3^, with rapidly growing interest in the fields of laser physics and ultrafast photonics^4–10^. From a fundamental perspective, an area of particular promise is the use of data-driven discovery to study nonlinear systems where determining an underlying governing physical model often proves elusive. The application of such inverse-problem like methods is motivated by the fact that although approaches using neural networks can yield accurate input-output descriptions of complex systems^11–16^, they do not provide any analytic framework with which to interpret the underlying physics. To this end, however, a number of reverse-engineering algorithms have recently been developed to identify the underlying mathematical structure of a system based only on analysis of data generated by the system. These results have naturally attracted much interdisciplinary attention, and have already been applied to many different physical problems^17–21^.

One particular approach to data-driven discovery aims to determine the smallest number of terms from a large library of potential candidate functions that can accurately represent a given data set via a system of coupled differential equations^22–25^. The methodology here is based on the empirical observation (from many areas of science) that the behaviour of even highly-complex systems is often governed by the interaction between a small number of distinct physical processes. This observation then allows the use of sparse regression to determine a model that is “parsimonious” i.e. containing the smallest number of terms capable of reproducing the observed behaviour without the presence of unnecessary overfitting^26,27^. The technique has become widely referred to as sparse identification of nonlinear dynamics (SINDy), and has been successfully applied in fields including chaotic systems, mechanics, hydrodynamics, and plasma physics^22,28^. In the field of nonlinear optics, its application has been more limited, although some studies have been reported identifying driving terms in soliton dynamics^29^, and mitigating impairments in telecommunication networks^30^. It is clear, however, that there is tremendous potential for much broader use of such techniques in optics.

In this paper, we report the first application of SINDy to study ideal optical four-wave mixing (FWM) in a nonlinear Schrödinger equation (NLSE) system^31–34^. In particular,﻿ using the approach introduced in Ref. ^22^ and the numerical toolbox in Ref. ^24^, we apply SINDy to analyse the interaction between a single frequency pump and two frequency detuned sidebands where the dynamics can be described by a reduced system of coupled differential equations. Using simulations to generate data from the system, we successfully recover the underlying physical model, both in the ideal noise-free case as well as in the presence of noise. For input data with noise, we discuss how spurious terms arising from overfitting can be identified such that the underlying model with the smallest number of terms can be determined. Although the ultimate aim of SINDy-like techniques is to discover physical models from experimental data, our results represent an important step in showing experimental feasibility. Indeed, since the FWM process underpins all Kerr-mediated CW wave-mixing phenomena, FWM is a highly representative test case for the use of SINDy in nonlinear optics. Moreover, although we consider the particular case of optical fibre dynamics, the NLSE is central to a wide range of other systems including cold atoms, plasma physics and hydrodynamics^35–38^, and we would expect these results to be readily transferrable to the study of FWM in these other systems.

## Summary of background theory

Four-wave mixing is a central process in nonlinear fibre optics, but its observation in isolation has been problematic because the wave mixing processes cascade with distance to generate multiple higher-order frequency components or sidebands^39–43^. The development of new experimental techniques, however, has recently allowed fibre FWM to be excited under close to ideal conditions^31,44,45^, providing new possibilities to study ideal wave mixing dynamics in the laboratory.

We consider here the particular case of degenerate FWM where two waves at a pump frequency undergo dynamical energy exchange with two equispaced sidebands. This is the system most often encountered experimentally^42^. We write the dimensionless evolving field as $A(\xi , \tau ) = A_{0}(\xi ) + A_{1}(\xi )\exp (i \Omega \tau ) + A_{-1}(\xi )\exp (-i \Omega \tau )$, where $A_0$ is the field at the pump frequency and $A_{\pm 1}$ are frequency detuned sidebands at ${\pm \Omega }$. Note that with $|A_{\pm 1}| \ll |A_{0}|$, the phase-matching condition for maximum FWM gain is $\Omega = \omega _{0} = \sqrt{2}$ which is the frequency condition we use here. However, similar results are obtained across the full range of gain $0< \Omega < 2.$

The dynamics of the field components are described by three coupled differential equations which can be reduced using a Hamiltonian formalism^46^ to a simpler system of two equations describing relative sideband intensity $\eta$ and phase $\phi$:

1a $$\begin{aligned} \frac{{{d}}\eta }{d{{\xi }}}= & {} 2 \eta ^{2} \sin \phi - 2 \eta \sin \phi \end{aligned}$$

1b $$\begin{aligned} \frac{{{d}}\phi }{d{{\xi }}}= & {} -(\Omega ^2+1) - 2 \cos \phi + 3 \eta +4 \eta \cos \phi . \end{aligned}$$

**Figure 1:**
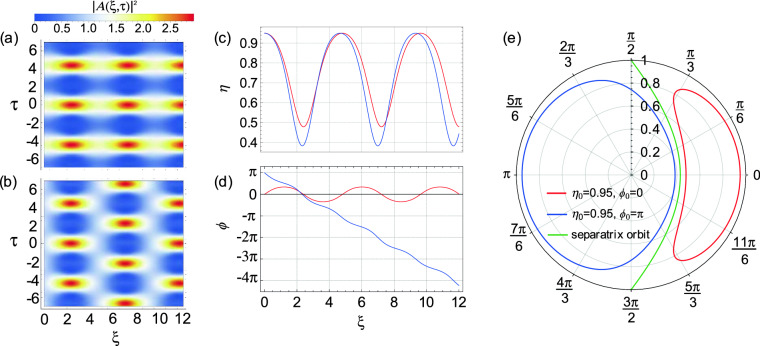
Spatio-temporal intensity evolution of ideal FWM for initial relative amplitude $\eta _0=0.95$ and different values of initial relative phase: (**a**) $\phi _0 = 0$, (b) $\phi _0 = \pi$. The transverse phase shift in the spatio-temporal evolution is apparent in (**b**). Subfigures (**c**) and (**d**) show respectively the corresponding evolution of $\eta (\xi )$ and $\phi (\xi )$, and (**e**) shows the corresponding phase-space portraits. The red curves in all subfigures are associated with initial condition $\phi _0 = 0$ and the blue curves with initial condition $\phi _0 = \pi$. The green curve in (**e**) represents the separatrix. The labelled vertical axis in (**e**) displays the range of $\eta$ over 0–1, whilst angles $\phi$ are shown around the circle.

Here $\eta = |A_{0}(\xi )|^{2}/(|A_{0}(\xi )|^{2} + |A_{-1}(\xi )|^{2} + |A_{1}(\xi )|^{2})$, and $\phi = \arg \big [A_{1}(\xi )\big ] + \arg \big [A_{-1}(\xi )\big ]- 2 \arg \big [A_{0}(\xi )\big ]$. In what follows, we assume equal initial sideband amplitudes $A_{1}(0) = A_{-1}(0)$ for convenience. (See the Methods sections for details of the coupled mode equations for the field components *A*, as well as the dimensional transformations in terms of the usual dispersion and nonlinearity parameters of nonlinear fibre optics.) This system can be readily solved numerically, and Fig. 1 shows false color plots of the spatio-temporal evolution of the intensity $|A(\xi ,\tau )|^2$ for initial conditions with identical relative amplitude but different initial relative phase: (a) $\eta _0 = 0.95, \, \phi _0 = 0$, and (b) $\eta _0 = 0.95, \, \phi _0 = \pi$. Both cases exhibit periodic spatio-temporal dynamics, but it is clear that the ($\pi$) out-of-phase initial condition in Fig. 1b introduces a phase-shift in the recurrence pattern. Figure 1c and d show respectively the associated differences in the evolution of $\eta (\xi )$ and $\phi (\xi )$, and Fig. 1e plots the dynamical orbits (phase-space portraits^46^) using $\eta (\xi )-\phi (\xi )$ polar coordinates. The red curves are associated with $\phi _0 = 0$ and the blue curves with $\phi _0 = \pi$. We note here that the FWM phase-space structure is well-known to be divided into two broad physical classes of dynamics on either side of a “separatrix,” shown as the green line in the figure. The separatrix boundary distinguishes qualitatively different regimes of spatio-temporal evolution depending on the presence (left hand side) or the absence (right hand side) of the transverse shift in spatio-temporal recurrence^44,45^. The use of the polar representation in Fig. 1e clearly illustrates how different orbits on each side of the separatrix are associated with different initial conditions. The separatrix itself is a limiting case associated with initial conditions $(\eta _0, \phi _0 ) \rightarrow (1,\pi /2)$, and leads physically to dynamics that are localized rather than periodic along the propagation dimension^44,47^. In fact, in the more general case when we are not limited to only four interacting waves, the separatrix trajectory describes the evolution of the Akhmediev breather^47,48^.

**Figure 2:**
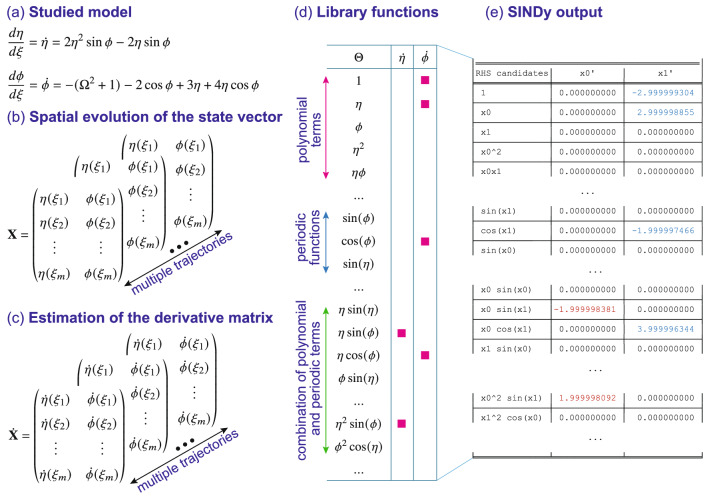
Illustration of SINDy applied to the analysis of FWM dynamics. (**a**) the ideal FWM system under study (in Hamiltonian form). (**b**) Multiple state vectors for different initial conditions. (**c**) Corresponding derivative matrices. (**d**) A selection of the 32 potential candidate library functions, with the red squares indicating those associated with the ideal FWM system. (**e**) Typical output showing the coefficients determined for each candidate function where output variables x0 and x1 correspond to $\eta$ and $\phi$ respectively. Nonzero coefficients for $\eta$ and $\phi$ are shown in red and blue respectively. Note that these particular results are obtained for noise-free data as discussed in the text.

The aim of SINDy is to determine the underlying dynamical system in Eq. (1) based only on generated data, and with minimal assumptions about the underlying physical model. Note here that the technique can of course be directly applied to the coupled mode equations for the field components (see Methods), but it is more convenient to analyse the relative amplitude and phase data, as these can be more readily measured^31^. Figure 2 illustrates SINDy applied to FWM. We study the evolution with distance $\xi$ of two variables: $\eta$ and $\phi$, and our aim is to invert a series of test data in these variables that has been numerically generated from the FWM system in Eq. (1). The input ${\mathbf {X}}$ consists of vectors $\eta (\xi _m)$ and $\phi (\xi _m)$ sampled at discrete $\xi _m$, and generated for multiple initial conditions (trajectories) as illustrated in Fig. 2b. After estimating the corresponding derivative matrices (Fig. 2c), a thresholded least squares algorithm attempts to identify the contributing terms that drive the evolution of the sampled $\eta (\xi _m)$ and $\phi (\xi _m)$. The terms are selected from a library (Fig. 2d) of 32 different functions: polynomials up to 3rd order, trigonometric functions of both variables, as well as their combinations: $\Theta (\varvec{\eta ,\phi }) = [1,\eta ,\phi ,\eta ^{2},\eta \phi , ..., \sin {\eta },\sin {\phi },\cos {\eta },...,\eta \sin {\eta },\eta \sin {\phi },\phi \cos {\eta },...,\eta ^{2} \sin {\eta },\phi ^{2} \sin {\phi },...]$. The choice to include both polynomial and trigonometric functions is based on how we might expect this system to evolve displaying characteristic Fermi-Pasta-Ulam periodic recurrence dynamics^41,42,48^. However, there is no *a priori* weighting attached to any of the library functions. The red squares in Fig. 2d highlight the “target” terms associated with the ideal FWM system.

## Results

**Input data with no noise**

We first consider noise-free data input. In particular, Eq. (1) is used to simulate 20 amplitude and phase trajectories $\eta$ and $\phi$ for different initial conditions ($\eta _{0}$,$\phi _{0}$) driving dynamics on both sides of the separatrix. Initial ($\eta _{0}$,$\phi _{0}$) are selected from uniform random distributions over the ranges [0,1] and [0,$2\pi$] respectively. We generate data with 12000 points in $\xi$ out to a maximum span of $\xi =12$, which typically contains $\sim$1-4 dynamical cycles (depending on the initial condition.)

Applying the SINDy algorithm to this noise-free data returned the exact form of the initial differential equation system Eq. (1), identifying both the dominant physical terms and the correct coefficients to an accuracy of $\sim \!10^{-6}$. This can be seen in Fig. 2e where we reproduce the raw algorithm output showing the coefficients returned for a selection of potential candidate functions. Coefficients of zero are returned when SINDy finds that the terms do not contribute to the dynamics. In fact, with the noise-free data, additional tests showed that we continue to obtain such precision in the inferred coefficients even using only 5 random trajectories.

**Figure 3:**
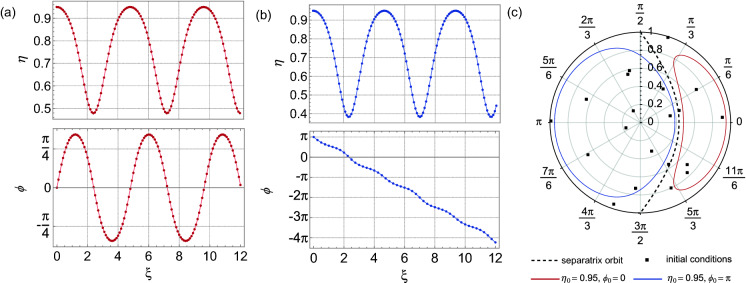
Dynamics reconstructed from the model returned by SINDy when analysing noise-free data. (**a**) Evolution of $\eta (\xi )$ and $\phi (\xi )$ for initial conditions $\eta _{0} = 0.95$, $\phi _{0} = 0$ from the returned model (circles) and the ideal model (solid line). (**b**) Evolution of $\eta (\xi )$ and $\phi (\xi )$ for initial conditions $\eta _{0} = 0.95$, $\phi _{0} = \pi$ from the returned model (circles) and the ideal model (solid line). (**c**) The corresponding dynamics plotted in $\eta -\phi$ polar coordinates. The black squares show the random initial conditions in the phase space that were used in this analysis. The labelled vertical axis in (**c**) displays the range of $\eta$ over 0-1, whilst angles $\phi$ are shown around the circle. Both the $\xi$-evolution and phase-space plots use red and blue line colors for dynamics on the right-hand side and left-hand side of the separatrix respectively (the separatrix is shown as the black dashed line). The threshold here was $\lambda =0.5$.

For completeness, Fig. 3a and b show the dynamics of $\eta (\xi )$ and $\phi (\xi )$ for two particular initial conditions on either side of the separatrix, both from the returned model (circles) and the ideal model (solid line). The results are visually indistinguishable. Figure 3c shows the corresponding phase space dynamics. The black squares here show the random initial conditions ($\eta _{0}$,$\phi _{0}$) used to generate the input data. In the absence of noise, the excellent agreement between returned and ideal model is perhaps not surprising given the known ability of SINDy to analyse chaotic systems such as the Lorenz equations^22^. However, this application to FWM clearly reveals how well the technique works where the system terms are periodic functions and not just simple polynomials.

**Input data with noise**

A more stringent test is model discovery from noisy data, as this points to experimental application. A problem, however, is that sparse regression is known to be potentially sensitive to noise, and it is therefore necessary to adapt techniques such as SINDy^49–51^. To this end, one approach has been to apply the SINDy algorithm separately to random subsets (“bootstraps”) of a given sequence of input data, thus yielding a number of distinct returned “models,” each associated with its own terms and coefficients^50^. The statistical analysis of these different coefficients then yields an estimation of mean values and uncertainties of the different contributing terms of the system.

In our analysis of noisy FWM data, we use a similar approach. However, instead of analysing bootstrapped samples from a single data series^50^, we consider an ensemble of input data based on scanning over different initial conditions; this would apply more typically to experiments in optics where it is straightforward to measure large data sets^31^. Specifically, we consider a total of 2000 simulated trajectories for random initial conditions ($\eta _0,\phi _0)$, and after computation of each trajectory, we apply random multiplicative Gaussian noise, with relative noise coefficient $\alpha$ (interpreted as a percentage) applied to the root mean-squared deviation of the data^50^. We then group the trajectories into 100 sets of 20 which are analysed by SINDy separately, returning 100 separate models, each with their own terms and coefficients.

**Figure 4:**
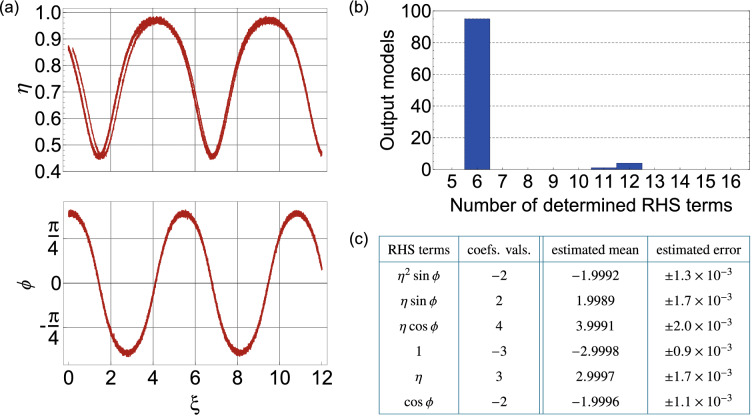
Input data and results returned by SINDy when analysing data with 2.5% noise. (**a**) Typical noisy data input to SINDy showing $\eta (\xi )$ and $\phi (\xi )$ evolution for initial conditions $\eta _{0} = 0.87$, $\phi _{0} = 1.2$ (**b**) Histogram showing the number of returned terms from applying SINDy to 100 data sets. (**c**) Computed mean and standard deviation of the coefficients of the 6-term models, compared with values expected from the ideal system in Eq. (1). The threshold here was $\lambda =0.5$.

Figure 4 shows results for the case of noisy FWM data with $\alpha = 2.5\%$. Firstly, to illustrate the level of noise on the input data, Fig. 4a plots $\eta$ and $\phi$ evolution for one set of initial conditions. To interpret the multiple results obtained using this approach, we first inspect a histogram of the number of non-zero terms associated with the 100 returned models as shown in Fig. 4b. For this case, 95 of the models possess only 6 terms, although some (overfitted) are returned possessing up to 12 terms (blue bars). For the 6-term models, we find that the terms are all identical, although the associated coefficients do vary. Aside from the fact that the 6-term model is the most frequently returned, this is also the natural choice from a physical perspective, where we seek the smallest number of contributions to the equations. The next step in the analysis is to compute the mean and standard deviation of the coefficients returned from these 95 models, and these results are shown in the table in Fig. 4c. We compare the results with the values expected from the ideal system in Eq. (1), and all coefficients are returned within 1 standard deviation. All standard deviations are at the $10^{-3}$ level.

**Figure 5:**
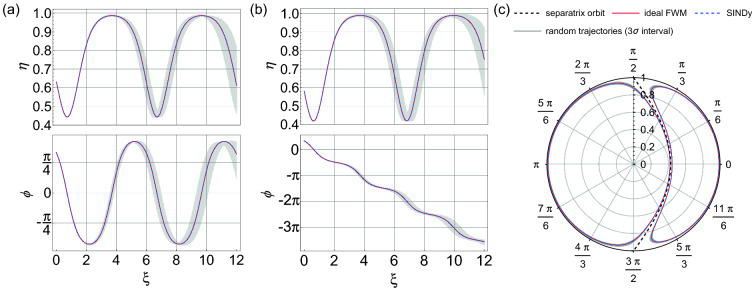
For input data with 2.5% noise, the figure shows the dynamics computed from the average model when the coefficients vary over their statistical range within three standard deviations. Results are shown for two initial conditions on either side of the separatrix: (**a**) $\eta _0 = 0.63, \phi _0 = \pi /3$, and (**b**) $\eta _0 = 0.58, \phi _0 = \pi /3$. The red curves shows the ideal dynamics expected from Eq. (3) which are visually indistinguishable from the blue dashed curves which plot the dynamics using the mean coefficients. The gray curves show the ensemble of potential dynamics by randomly sampling the model coefficients within their three standard deviation errors. (**c**) The corresponding results plotted in phase space, with the labelled vertical axis displaying the range of $\eta$ over 0–1, whilst angles $\phi$ are shown around the circle. The black dashed line shown in this plot is the separatrix.

Figure 5 shows additional tests of the accuracy of the “mean model” computed over the ensemble. Firstly, for initial conditions on either side of the separatrix: (a) $\eta _0 = 0.63, \phi _0 = \pi /3$, and (b) $\eta _0 = 0.58, \phi _0 = \pi /3$, Fig. 5a and b show amplitude and phase evolution from the mean model (blue dashed curves) compared with the ideal model from Eq. (1) (red curves). The results are visually indistinguishable. Note that these particular initial conditions are selected because the orbits lie close to the separatrix and are thus the most challenging to correctly reconstruct. We can also test how the mean model predicts dynamics when the coefficients are varied over their statistical uncertainty limits. Randomly sampling the model coefficients within a range of three standard deviations generates the ensemble of potential dynamics shown as the gray curves in the figure. The corresponding results plotted in phase space are shown in Fig.  5c.

**Figure 6:**
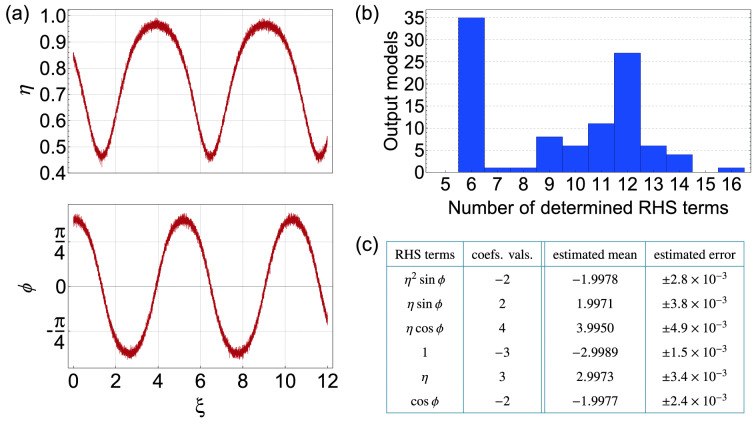
Input data and results returned by SINDy when analysing data with 5% noise. (**a**) Typical noisy data input to SINDy showing $\eta (\xi )$ and $\phi (\xi )$ evolution for initial conditions $\eta _{0} = 0.84$, $\phi _{0} = 1.16$ (**b**) Histogram showing the number of returned terms from applying SINDy to 100 data sequences. (**c**) Computed mean and standard deviation of the coefficients of the 6-term models, compared with values expected from the ideal system in Eq. (3). The threshold here was $\lambda =0.5$.

**Figure 7:**
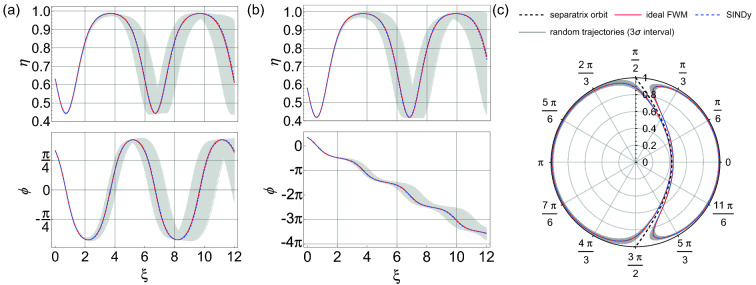
For input data with 5% noise, the figure shows the dynamics computed from the average model when the coefficients vary over their statistical range within three standard deviations. Results are shown for two initial conditions on either side of the separatrix: (**a**) $\eta _0 = 0.63, \phi _0 = \pi /3$, and (**b**) $\eta _0 = 0.58, \phi _0 = \pi /3$. The red curves shows the ideal dynamics expected from Eq. (3) which are visually indistinguishable from the blue dashed curves which plot the dynamics using the mean coefficients. The gray curves show the ensemble of potential dynamics by randomly sampling the model coefficients within their three standard deviation errors. We set $\lambda =0.5$ in these simulations. (**c**) The corresponding results plotted in phase space. The labelled vertical axis displays the range of $\eta$ over 0–1, whilst angles $\phi$ are shown around the circle. The black dashed line shown in this plot is the separatrix.

Figures 6 and 7 show similar results to those in Figs. 4 and 5, but for increased noise of $\alpha = 5\%$, and again analysing 100 sets of 20 trajectories for random initial conditions. The higher level of noise on the input data is clear from Fig. 6a, and the histogram in Fig. 6b shows how this leads to a qualitatively different output. The histogram reveals a broader range of returned models (up to 16 terms), but at the same time, it also shows a clear peak associated with only 6 terms. Significantly, when analysing these results in more detail, the 35 returned 6-term models in this case all give the same terms as in the ideal model, and these results are shown in the table in Fig. 6c. When we compute the mean coefficients, the standard deviations are still in the range $\sim \!10^{-3}$ although, as might be expected, they are larger than in the case of lower noise (compare with Fig. 4c). For the same initial conditions as Figs. 5, 7 shows the system dynamics from the mean model, and again the dynamics computed using the mean coefficients (blue dashed curves) are visually indistinguishable from the ideal dynamics (red curves). We also study the dynamics from the model when the coefficients vary over their uncertainty limits and here, the multiple trajectories (grey curves) show a greater variation than for the lower noise case in Fig. 5.

At an even higher noise level of $\alpha = 7.5$%, a similar histogram to that in Fig. 6 was obtained, with the 6-term model still being that most frequently returned. However, the values of the associated coefficients varied more significantly, with differences compared to the expected ideal values at the $\sim \!10^{-2}$ level. Although satisfactory from the perspective of model discovery, this is an order of magnitude higher that with the lower noise levels above. It is also interesting to note that when we examine the overfitted models with more than 6 terms in this case, the computed coefficients are typically associated with large standard deviations (in some cases exceeding 100% of the mean values) and the trajectories computed over the coefficient uncertainties diverge from the expected trajectories after the initial stage of propagation (typically after one recurrence cycle.) At an even higher level of noise of $\alpha = 10$%, the histogram distribution became essentially uniform, and it was no longer possible to reliably say that any particular model was most frequently returned.

## Discussion and conclusions

The main result of this study is that we have shown that data driven discovery using sparse regression (SINDy) can indeed successfully determine the governing model for nonlinear four-wave mixing. The fact that this is possible in the absence of noise is expected based on previous studies^22^, but our results also show successful results at noise levels of 5% which are likely to be obtainable in experiments^31^. Of course, the physics of FWM is well-known, but our aim is to demonstrate the feasibility of this technique, with the ultimate objective being to use it to analyse data from a nonlinear fibre system where the underlying model is not known in advance. In addition, in demonstrating the success of SINDy with FWM, we anticipate that it will be readily adapted to work with similar systems of coupled equations in optics describing e.g. multiple pump parametric amplification, CW Raman scattering etc^42^. Overall, this work represents a further step in showing the feasibility of data-driven discovery in nonlinear optics.

A further significant result in this paper is the development of a useful approach to interpret the results of SINDy in the presence of noise by analysing an ensemble of input data computed over different initial conditions spanning the dynamical phase space. This involves inspection of a histogram distribution of the number of terms associated with multiple returned models, followed by computation of mean and uncertainty in the associated term coefficients. This allows us to readily assess the predictive accuracy of the model by computation of the dynamics within the term uncertainty limits. We also note here that sampling over multiple initial conditions is advantageous in exploring the dynamical space more completely when compared to performing repeated sampling of only one set of initial conditions. Moreover, our results suggest that when a nonlinear system contains a separatrix boundary between qualitatively different dynamics, sampling initial conditions on both sides of the separatix is necessary for SINDy to robustly return the underlying model. Indeed, when we used input data evaluated only over a small subset of initial conditions (a small localised region of the phase space) SINDy returned models with fewer than 6 terms, which is clearly not the desired physical description of FWM. There are also improvements that one can consider to our approach such as combining an ensemble over initial conditions with internal data bootstrapping within each data set. In addition, our analysis here has not implemented any specific preprocessing step to improve the calculation of numerical derivatives, and this is also a natural area of future work^25^.

It is of course important to note that not all nonlinear processes in optics can be described by coupled equations suitable for analysis using SINDy. In particular, the most general modelling of nonlinear propagation in optical fibre (including soliton effects and processes such as self- and cross-phase modulation) is the generalised nonlinear Schrödinger equation, a partial differential equation that includes multiple derivative terms describing higher order dispersion, instantaneous and delayed nonlinear response, and dissipation^42^. However, although the basic technique of SINDy is not appropriate in this case, several extended methods of sparse identification have been developed and indeed enable model discovery resulting from partial differential equation dynamics^23,29,52^.

As a more general conclusion, it is clear that sparse regression using SINDy promises to be a very powerful technique amongst the toolbox of methods available to researchers in nonlinear optics. In this context, we stress that SINDy does not aim to provide a “black box” system description (such as might be provided by a neural network), but rather it should always be used in parallel with consideration of the underlying physics. Indeed, it would be expected that any model(s) returned by SINDy for an unknown system would be accompanied by parallel analysis to guide the search for an appropriate and physically-justified theoretical description. Of course, with the overall objective being the analysis of a partially-understood system, a key element is the need to develop strategies to distinguish between different models that may be returned. We anticipate that the results presented here may point to further research in this direction.

## Methods

### Theory of ideal FWM

Four-wave mixing in optical fibre has been studied extensively, from the first days of nonlinear optics to recent applications developing broadband frequency combs^42^. The fundamental propagation model is the nonlinear Schrödinger equation which is written in normalised form:

2 $$\begin{aligned} i\frac{\partial A}{\partial \xi } + \frac{1}{2}\frac{\partial ^2 A}{\partial \tau ^2} + |A|^2 A = 0. \end{aligned}$$

Here, dimensionless propagation distance and time are defined as: $\xi = z/L_{NL}$, $\tau = t/\sqrt{|\beta _{2}|L_{NL}}$, where $L_{NL} = (\gamma P_0)^{-1}$. Here $P_{0}$ is power and $\beta _2$ and $\gamma$ are the usual dimensional fiber dispersion and nonlinearity parameters respectively^42^. The dimensionless field envelope $A(\xi , \tau )$ is normalized with respect to $P_{0}^{1/2}$.

The theoretical model for ideal FWM^46,47^ writes the evolving field in the optical fibre in the form: $A(\xi , \tau ) = A_{0}(\xi ) + A_{1}(\xi )\exp (i \Omega \tau ) + A_{-1}(\xi )\exp (-i \Omega \tau )$, where $A_0$ is the field at the pump frequency and $A_{\pm 1}$ are the frequency detuned sidebands at ${\pm \Omega }$. The fields $A_0$ and $A_{\pm 1}$ are complex-valued. This case corresponds to degenerate FWM with two waves at the pump frequency. The dynamics of the field components are then given by the coupled mode equations:

3 $$\begin{aligned} \begin{aligned} -i\frac{{{d}}A_{0}}{d{{\xi }}}&= \Big (|A_{0}|^{2} + 2\big [|A_{-1}|^{2}+ |A_{1}|^{2}\big ] \Big )A_{0} + 2A_{-1}A_{1}A_{0}^*\\ -i\frac{{{d}}A_{-1}}{d{{\xi }}}&= \Big (-\frac{1}{2}\Omega ^2 +|A_{-1}|^{2} + 2\big [|A_{0}|^{2}+ |A_{1}|^{2}\big ] \Big )A_{-1} + A_{1}^*A_{0}^2 \\ -i\frac{{{d}}A_{1}}{d{{\xi }}}&= \Big (-\frac{1}{2}\Omega ^2 + |A_{1}|^{2} + 2\big [|A_{-1}|^{2}+ |A_{0}|^{2}\big ]\Big )A_{1} + A_{-1}^*A_{0}^2 \end{aligned} \end{aligned}$$

Note that with $|A_{\pm 1}| \ll |A_{0}|$, the phase-matching condition for maximum FWM gain is $\Omega = \omega _{0} \sqrt{|\beta _{2}|/\gamma P_{0}} = \sqrt{2}$, a result that is also readily derived from a modulation instability analysis. Note that all the results in this paper correspond to this condition, but similar results are obtained across the full range of gain $0< \Omega < 2.$ The Hamiltonian representation given in Eq. (1) is derived from these coupled amplitude equations by defining real variables describing the relative sideband intensity $\eta$ and phase $\phi$, where $\eta = |A_{0}(\xi )|^{2}/(|A_{0}(\xi )|^{2} + |A_{-1}(\xi )|^{2} + |A_{1}(\xi )|^{2})$, and $\phi = \arg \big [A_{1}(\xi )\big ] + \arg \big [A_{-1}(\xi )\big ]- 2 \arg \big [A_{0}(\xi )\big ]$. We assume equal initial sideband amplitudes $A_{1}(0) = A_{-1}(0)$ throughout the paper.

The FWM relative amplitude and phase data for input to the SINDy algorithm is generated from the numerical integration of the coupled equations in Eq. (1) using standard numerical methods^53,54^. The input ${\mathbf {X}}$ to SINDy consists of vectors $\eta (\xi _m)$ and $\phi (\xi _m)$ sampled at discrete $\xi _m$, and generated for multiple initial conditions to fully sample the phase space on both sides of the separatrix (see the black squares in Fig. 3c). The integration relative and absolute tolerance were both $\sim$10$^{-8}$. To generate the input datasets with added noise, we first integrated Eq. (1) as above, and then added Gaussian noise to the noise-free data.

### Description of SINDy

The SINDy technique^22^ considers a dynamical system of the form:

4 $$\begin{aligned} \frac{d}{d\zeta }{\mathbf {x}}(\zeta ) = {\mathbf {f}}\big [ {\mathbf {x}}(\zeta ) \big ]. \end{aligned}$$

Here the state vector is ${\mathbf {x}} = \big [x_{1}(\zeta ); x_{2}(\zeta ); ...; x_{n}(\zeta ) \big ]$, where the *n* variables $x_{1}(\zeta ) ... x_{n}(\zeta )$ correspond to measurable physical quantities of interest (e.g. amplitude, phase, intensity, displacement) which evolve as a function of a variable $\zeta$ (e.g. distance, time). The function ${\mathbf {f}}\big [ {\mathbf {x}}(\zeta ) \big ]$ describes the associated dynamical constraints. A data set describing the spatial or temporal evolution is represented in a matrix form ${\mathbf {X}} = \big [x_{1}(\zeta _{1},...,\zeta _{m});x_{2}(\zeta _{1},...,\zeta _{m});...;x_{n}(\zeta _{1},...,\zeta _{m}) \big ]$ sampling the *n* physical variables *x* at *m* discrete values of $\zeta$. The choice of the dimensionality of the state vector, the number of sampling points, and the sample spacing in the data set is linked to the physical problem under study. It is also possible that an extended data set consists of multiple trajectories of ${\mathbf {X}}$ corresponding to the system evolution with different initial conditions.

Based on the data set ${\mathbf {X}}$, the algorithm numerically estimates the derivatives to yield $\dot{{\mathbf {X}}}$, from which we are able to determine the underlying model from the equation:

5 $$\begin{aligned} \dot{{\mathbf {X}}} = \Theta ({\mathbf {X}}) {\mathbf {M}}. \end{aligned}$$

Here $\Theta ({\mathbf {X}})$ on the right-hand side (RHS) represents a library of potential candidate dynamical functions that act on the columns of ${\mathbf {X}}$, while ${\mathbf {M}} = \big [\varvec{\mu _{1}}, \varvec{\mu _{2}}, ..., \varvec{\mu _{n}} \big ]$ represents a row vector of associated coefficients. In general, the library may consist of any number of polynomial, periodic or other mathematical functions of ${\mathbf {X}}$ (and their combinations), but it is usually possible to limit the size of the library based on the expected physical properties of the system. The non-zero row vector coefficients ${\mathbf {M}}$ are estimated by inverting Eq. (5) via a sequential thresholded least-squares algorithm^22,52^, where the threshold parameter $\lambda$ specifies the minimum magnitude for possible returned coefficients: coefficients with magnitude lower than the threshold are zeroed during the algortithm iterations. The choice of threshold for any given problem depends on the form of the system being studied, and can be optimized empirically to favour convergence^52^. It is of course highly preferable to work with normalised data and equations such that all coefficients have comparable magnitudes. In what follows below, we typically found that the range $\lambda \!\sim 0.5-0.8$ yielded good results. The output returned by SINDy is an estimated representation of the dynamical model Eq. (4) which can be written as:

6 $$\begin{aligned} \dot{{\mathbf {x}}}(\zeta ) = {\mathbf {f}}\big [ {\mathbf {x}}(\zeta ) \big ] = {\mathbf {M}}^{T} \big [\Theta ({\mathbf {x}}^{T}(\zeta )) \big ]^{T}. \end{aligned}$$

This system contains the identified structure and coefficients of the different terms of the differential equations for each element of the state vector ${\mathbf {x}}$.

Figure 3 illustrates how SINDy is applied to the phase-space dynamical model of FWM. We are specifically interested in the evolution with distance $\xi$ of two variables: amplitude ($\eta = x_0$) and phase ($\phi = x_1$), and our aim is to invert a series of test data that has been numerically generated from the ideal system in Eq. (1). The algorithm input ${\mathbf {x}}$ consists of a sequence of vectors of $\eta (\xi _m)$ and $\phi (\xi _m)$ sampled at discrete $\xi _m$, and generated for multiple initial conditions (trajectories) as illustrated in Fig. 2b. After estimation of the derivative matrix for each trajectory (Fig. 2c), this data set is processed by the algorithm as described above. The library of potential candidate functions on the right-hand side of the unknown model Eq. (5) is illustrated schematically in Fig. 2(d) and includes a total of 32 different functions: polynomial functions (up to 3rd order), trigonometric (periodic) functions of both variables, as well as their combinations: $\Theta (\varvec{\eta ,\phi }) = [1,\eta ,\phi ,\eta ^{2},\eta \phi , ..., \sin {\eta },\sin {\phi },\cos {\eta },...,\eta \sin {\eta },\eta \sin {\phi },\phi \cos {\eta },...,\eta ^{2} \sin {\eta },\phi ^{2} \sin {\phi },...]$. Based on data, SINDy will attempt to estimate the contributing dynamical terms that underlie the evolution of the sampled $\eta (\xi _m)$ and $\phi (\xi _m)$. Figure 2d highlights the “target” terms associated with the ideal FWM system by red squares. Figure 2e illustrates the algorithm results for noise-free data which are output in terms of a matrix with both zero and non-zero coefficients.

Our implementation of SINDy used the publically-available open-source code^24,55^. For the main result of this paper (the Hamiltonian system) computation times on a standard Windows PC with 3.00 GHz 6 MB cache double-core CPU were as follows: 0.9 s for 20 trajectories of noise-free input data; 110.2 s for 100 sets of 20 trajectories for 2.5% noise (including the time spent for the statistical analysis of the returned models); 110.3 s for 100 sets of 20 trajectories for 5% noise (including the time spent for the statistical analysis of the returned models).

### SINDY applied to FWM amplitude equations

Although the results above consider the Hamiltonian system in Eq. (1) with 6 dynamical terms, we can also apply SINDy directly to the complex amplitude system of 3 differential equations in Eq. (3). To this end, we first write the complex amplitudes in terms of real and imaginary parts: $A_{0}(\xi ) = a_{0} + ib_{0}$ and $A_{1}(\xi ) = A_{-1}(\xi ) = a_{1} + ib_{1}$, and as above we assume initially equal sideband amplitudes such that the spatial evolution of $A_{1}(\xi )$ and $A_{-1}(\xi )$ is identical. This yields 4 coupled amplitude equations, but involving a total of 22 different dynamical terms:.

7a $$\begin{aligned} \frac{{{d}}a_{0}}{d{{\xi }}}= & {} -(a_{0}^{2} + b_{0}^{2} + 2a_{1}^{2} + 6b_{1}^{2})b_{0} - 4a_{0}a_{1}b_{1} \end{aligned}$$

7b $$\begin{aligned} \frac{{{d}}b_{0}}{d{{\xi }}}= & {} (a_{0}^{2} + b_{0}^{2} + 6a_{1}^{2} + 2b_{1}^{2})a_{0} + 4b_{0}a_{1}b_{1}\end{aligned}$$

7c $$\begin{aligned} \frac{{{d}}a_{1}}{d{{\xi }}}= & {} (\Omega ^{2}/2)b_{1} - \big (a_{0}^{2} + 3[b_{0}^{2} + a_{1}^{2} + b_{1}^{2}] \big )b_{1} - 2a_{0}b_{0}a_{1}\end{aligned}$$

7d $$\begin{aligned} \frac{{{d}}b_{1}}{d{{\xi }}}= & {} -(\Omega ^{2}/2)a_{1} + \big (b_{0}^{2} + 3[a_{0}^{2} + a_{1}^{2} + b_{1}^{2}] \big )a_{1} + 2a_{0}b_{0}b_{1}, \end{aligned}$$

To apply SINDy to this system, we create a library function of potential candidate terms $\Theta (a_{0},b_{0},a_{1},b_{1}) = [1,a_{0},b_{0},a_{1},b_{1}, ..., a_{0}^2,a_{0}b_{0},a_{0}a_{1},...,b_{1}^{3}a_{1}, b_{1}^{4}]$ containing polynomials of the four variables extended up to the quartic order yielding a total of 70 possible RHS terms. Although this is a significantly more complex case than the Hamiltonian system with only 6 terms, we followed a similar approach to that described above, first applying SINDY to noise-free data. Here, it successfully identified all the correct dynamical terms (to an accuracy of $\sim \! 10^{-5},$ with no overfitting). On the other hand, as might be expected, the larger number of potential terms in the system means that noise has a much greater effect. Indeed, obtaining uncertainties of $\sim \! 10^{-3}$ around the expected correct values of the coefficients of the 22 terms was only possible with an order of magnitude less noise of 0.25% compared to the results obtained with the Hamiltonian system. This result stresses the importance of combining SINDy with physical insight in order to construct the most useful model for a given problem.

## Data Availability

The data underlying the results presented in this paper are available from the authors upon reasonable request.
