# Author Correction: Data-driven model discovery of ideal four-wave mixing in nonlinear fibre optics

**DOI:** 10.1038/s41598-022-25523-5

**Published:** 2022-12-06

**Authors:** Andrei V. Ermolaev, Anastasiia Sheveleva, Goëry Genty, Christophe Finot, John M. Dudley

**Affiliations:** 1 Institut FEMTO-ST, Université Bourgogne Franche-Comté CNRS UMR 6174, 25000 Besançon, France; 2 Laboratoire Interdisciplinaire Carnot de Bourgogne, Université Bourgogne Franche-Comté CNRS UMR 6303, 21078 Dijon, France; 3 Photonics Laboratory, Tampere University, FI-33104 Tampere, Finland

Correction to: *Scientific Reports* https://doi.org/10.1038/s41598-022-16586-5, published online 26 July 2022

In the original version of the Article, Figure 4 (C) was a duplication of Figure 6 (C).

The original Figure 4 and accompanying legend appear below.

**Figure 4:**
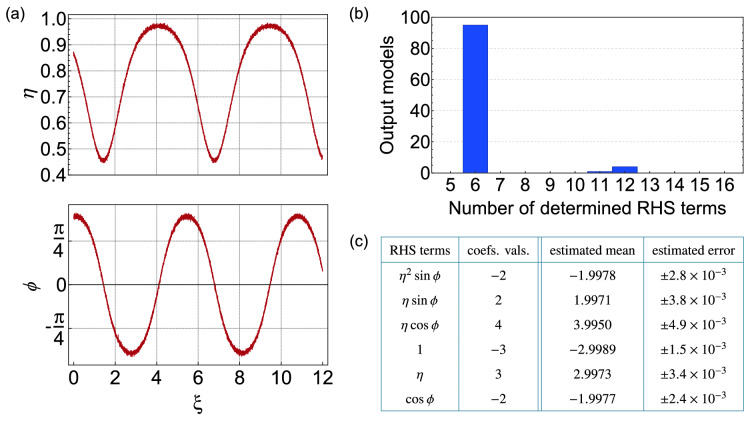
Input data and results returned by SINDy when analysing data with 2.5% noise. (**a**) Typical noisy data input to SINDy showing *η*(*ξ*) and *ϕ*(*ξ*) evolution for initial conditions *η*_0_ = 0.87, *ϕ*_0_ = 1.2 (**b**) Histogram showing the number of returned terms from applying SINDy to 100 data sets. (**c**) Computed mean and standard deviation of the coefficients of the 6-term models, compared with values expected from the ideal system in Eq. (1). The threshold here was *λ* = 0.5.

The original Article has been corrected.

